# Papillary thyroid cancer with Hashimoto’s thyroiditis attenuates the tumour aggressiveness through the up-regulation of E-cadherin and TGF-β expression

**DOI:** 10.1007/s10238-022-00857-6

**Published:** 2022-07-24

**Authors:** Su Jin Kim, Seung-Eun Lee, Young Il Kim, Il Sung Nam-Goong, Hyo Won Jung, Eun Sook Kim

**Affiliations:** 1 Department of Anesthesiology and Pain Medicine, College of Medicine, Dongguk University, Gyeongju, 38066 South Korea; 2 Department of Internal Medicine, Ulsan University Hospital, University of Ulsan College of Medicine, Ulsan, 44033 South Korea; 3 Department of Herbology, College of Korean Medicine, Dongguk University, Gyeongju, 38066 South Korea

**Keywords:** Papillary thyroid cancer, Hashimoto’s thyroiditis, E-cadherin, Transforming growth factor beta

## Abstract

Human papillary thyroid cancer (PTC) is often associated with Hashimoto’s thyroiditis (HT), and their coexistence improves the prognosis of PTC. *Aim of the study*. The objective of our study is to investigate the expression of cadherins and TGF-β which are regulators in the tumour aggressiveness with metastatic spread in PTC patients and its relationship with HT. The expression of E-cadherin and N-cadherin was measured in thyroid tissues of healthy volunteers and PTC patients with HT (PTC/HT) or without. The E-cadherin expression was also determined in thyroid cancer cells (TPC1, SNU373, SNU790, 8505C, CAL62, and FTC133). Cell migration was measured by wound healing assay. The expression of N-cadherin, ICAM1, and TGF-β was measured in thyroid tissues and plasma. The E-cadherin expression was significantly increased in PTC/HT patients compared with PTC alone. Meanwhile, the N-cadherin expression was significantly decreased in PTC/HT patients. The E-cadherin expression was only observed in FTC cells, and the overexpression of E-cadherin inhibited cancer cell migration. The TGF-β expression was significantly increased in PTC/HT patients, and the plasma levels were higher in PTC/HT patients than in PTC alone. The expression of N-cadherin and ICAM-1 was significantly decreased in PTC/HT patients. Our results indicate that the expression of E-cadherin and TGF-β was higher in PTC/HT patients than in PTC alone. This suggests that the presence of PTC with HT may attenuate the tumour aggressiveness and metastasis through the up-regulation of E-cadherin and TGF-β expression.

## Introduction

Thyroid cancer is one of the most common malignancies in the endocrine system. Papillary thyroid cancer (PTC) is the most prevalent manifestation of thyroid cancer with a 10-year relative survival rate of 93% [1]. In South Korea, the prevalence of PTC is rapidly increasing, accounting for > 95% of all thyroid cancer [2]. Hashimoto’s thyroiditis (HT) is a chronic inflammation of the thyroid gland and the most common cause of hypothyroidism in iodine sufficient areas of the world, with a particularly high prevalence among older individuals [3]. Although HT is known to increase the risk of PTC [4, 5], the presence of HT in patients with PTC is related with a less aggressive clinical presentation and low recurrence rate [6, 7]. Differentiated thyroid cancer including PTC usually expresses thyroid specific antigens as normal follicular epithelial cells. In this regard, the autoimmune response to thyroid-specific antigens in patients with HT, which may be involved in the destruction of cancer cells expressing thyroid-specific antigen in PTC patients, thus prevents recurrence and improves survival. However, the relationship between PTC and HT has remained controversial.

Cadherins are a group of type-1 transmembrane proteins that depend on calcium ions to function and can regulate cell–cell adhesion, forming adherent junctions to bind cells within tissues together [8]. Different members of the cadherin family are found in different locations, e.g. E-cadherin in epithelial tissue, N-cadherin in neurons, and P-cadherin in the placenta [9]. Some studies have shown aberrant expression of E-cadherin with the development of metastases in cancers including thyroid cancer [10, 12]. E-cadherin is a calcium-dependent intercellular adhesion molecule and is specifically involved in epithelial cell-to-cell adhesion [10]. In cancer, E-cadherin expression is often down-regulated during carcinoma progression and metastatic spread of aggressive tumours such as oesophagus, ovary, and stomach [11]. The selective loss of E-cadherin can cause dedifferentiation and invasiveness in human carcinomas, indicating E-cadherin to be a classic tumour suppressor [12]. Some studies in thyroid cancer have demonstrated that differentiated cancers with a poorer prognosis present reduced E-cadherin expression, but its expression does predict the extra-thyroidal growth of thyroid cancers [13].

Therefore, in this study, to investigate the relationship with HT in PTC patients on the tumour aggressiveness with metastatic spread, we examined the expression of E-cadherin and TGF-β in thyroid tissues with plasma and different types of thyroid cancer cells.

## Materials and methods

### Patient samples

Between April 2008 and July 2013, a total of 86 consecutive patients were referred and screened for the study. We ruled out other cancers and autoimmune disorders, diabetes mellitus, renal disorder, liver disorder, or any other inflammatory or medical condition which would have influenced the parameters under study. Blood was collected from patients before surgery and from healthy volunteers as normal controls. After all, a total of 76 patients were actually enrolled in our present study including 12 men and 64 women, with a mean age of 46 years. Thirteen were healthy controls, 48 had papillary thyroid cancer (PTC) alone, and 28 had PTC with Hashimoto’s thyroiditis. Tissue samples were obtained during thyroidectomies performed in the department of surgery at Ulsan University Hospital. After surgical resection, thyroid tissue samples were prepared by a pathologist who selected tumour and normal tissues.

From the surgical specimens of these patients, a total of 34 tissue samples were collected, including 17 PTC tissue samples and 17 PTC with HT tissue samples. Thyroid tissue samples were frozen in liquid nitrogen and stored at − 70 °C. Tumour staging was based on the recommendations of the seventh edition of the American Joint Committee on Cancer (AJCC).

### RNA extraction and reverse transcription-polymerase chain reaction

Total RNA from normal or cancer tissues of PTC and PTC with HT samples was prepared using the TRI reagent (Sigma-Aldrich, St Louis, MO, USA). Approximately 1 µg of total RNA was converted into cDNA using the AccuScript™ high-fidelity 1^ST^ strand cDNA synthesis kit (Stratagene, Santa Clara, CA, USA) at 65 °C for 5 min, 42 °C for 1 h, and 70 °C for 15 min. For the PCR reaction, 10 × buffer, 10 mM dNTP mix, Taq polymerase (New England Biolabs, Ipswich, MA, USA), and specific primers (Table 1) were mixed with cDNA. PCR products were subjected to 1.5% agarose gel electrophoresis and visualised under UV light. Images were acquired using the Bio-Rad Molecular Imager ChemiDoc XRS (Bio-Rad, Hercules, CA, USA).

**Table 1:** Sequences of the primers for target genes

Primers	Sequence (5′ → 3′)
*E-cadherin*	
Forward	CTTCACCGACTTACCTACT
Reverse	GTGCCATACACTTAATTCTC
*N-cadherin*	
Forward	TCAGTGGCGGAGATCCTACT
Reverse	GTGCTGAATTCCCTTGGCTA
*TGF-β1*	
Forward	CGCGTGCTAATGGTGGAAA
Reverse	CGGAGCTCTGATGTGTTGAA
*DKK3*	
Forward	ACAGCCACAGCCTGGTGTA
Reverse	CCTCCATGAAGCTGCCAAC
*ICAM1*	
Forward	AAATCTGGCACCACACCTTC
Reverse	TGATCTGGGTCATCTTCTCG
*β-actin*	
Forward	AGAGCTACGAGCTGCCTGAC
Reverse	AGCACTGTGTTGGCGTACAG

### Real-time PCR

The cDNA from normal, PTC, and PTC with HT samples was used for real-time PCR using SYBR Green. The expression of genes was normalised using β-actin as a housekeeping gene. Real-time PCR was carried out using C1000 Thermal Cycler **(**Bio-Rad, Hercules, CA, USA). The following primers were used for the amplification of ICAM1 and β-actin: ICAM1 forward, 5′-CCTATGGCAACGACTCCTTCT-3′ and reverse, 5′-GTCTGCAGTGTCTCCTGGCTC-3′; β-actin forward, 5′**-**AAATCTGGCACCACACCTTC**-**3′ and reverse, 5′**-**TGATCTGGGTCATCTTCTCG-3′. Calculations were made based on the values of the cycle threshold (ΔCt), which was determined by normalising the average Ct value of each treatment to that of the β-actin control and then calculating 2^−ΔΔCt^ for each treatment.

### Western blot analysis

Total protein extracts from normal, PTC, and PTC with HT samples were prepared using the T-PER tissue protein extraction reagent (Pierce, Rockford, IL, USA) containing a cocktail of protease inhibitors (Pierce, Rockford, IL, USA). Protein concentration was determined with the BCA protein assay kit (Pierce, Rockford, IL, USA) according to the manufacturer’s protocol. Proteins were separated by SDS-PAGE in 6–12% polyacrylamide gels and transferred onto nitrocellulose membranes (Millipore Corp, Bedford, MA, USA). Membranes were blocked with Tris-buffered saline with 0.1% Tween 20 (TBST) containing 5% non-fat dry milk for 60 min at room temperature. After washing four times with TBST, membranes were incubated with primary antibodies for E-cadherin, pSTAT3 (Cell Signaling Technology, Danvers, MA, USA), TGF-β1, and VEGF (Santa Cruz Biotechnology, Santa Cruz, CA, USA) at 4 °C overnight, washed four times with TBST, and incubated with horseradish peroxidase-conjugated species-specific secondary antibodies (Santa Cruz Biotechnology, Santa Cruz, CA, USA) for 1 h at room temperature and then washed again four times. Signals were visualised using the Bio-Rad Molecular Imager ChemiDoc XRS (Bio-Rad, Hercules, CA, USA) with the Immun-Star™ Western™ Chemiluminescent kit (Bio-Rad, Hercules, CA, USA).

### Cell culture

All human thyroid cancer cells (TPC1, FTC, SNU373, SNU790, 8505c, and CAL62) were kindly provided by Dr YK Shong, Department of Internal Medicine, Asan Medical Center, College of Medicine University of Ulsan, Korea. The cells were cultured on tissue culture dishes (Falcon, San Jose, CA, USA) in Dulbecco's Modified Eagle's Medium (DMEM) or RPMI-1640 medium containing 10% foetal bovine serum (FBS). For culture, 5 × 10^5^ cells were cultured at 37 °C in a humidified atmosphere containing 5% CO_2_.

### Transfection of E-cadherin

TPC1 cells were seeded in 6-well plates and transfected with E-cadherin or control (pCMV/hygro) vector (Sino Biological Inc., Beijing, China). Transfection was carried out using FuGENE HD transfection reagent (Roche, Mannheim, Germany) according to the manufacturer’s protocol. After transfection, cells stably transfected with human E-cadherin were selected by adding hygromycin (Invitrogen).

### Wound healing assay

TPC1, FTC 133 cells, and control plasmid (TPC1/CON) or E-cadherin expressing TPC1 (TPC1/E-cadherin) cells (5 × 10^4^ cells/well) were seeded in 6-well plates for 24 h. After reaching from 70 to 80% confluence, a micropipette tip was used to the scratch cells. After observing the wound created by scratching the cellular monolayer, fresh media were applied and photographic imaging was performed using a microscope.

### Enzyme-linked immunosorbent assay

Plasma samples obtained from patients and healthy controls and stored at − 70 °C were used for quantification of TGF-β1 concentration by enzyme-linked immunosorbent assay (ELISA) (Duo Set ELISA kit, R&D Systems, Minneapolis, MN, USA) according to the manufacturer’s instructions. Briefly, 50 μL of assay diluent was added to each well and subsequently, standard, control, or plasma samples were added and incubated for 2 h at room temperature. After aspiration of each well and four washes with wash buffer, 100 µL of conjugate was added to each well, incubated for 2 h, and the last washing step was repeated. Substrate solution was then added to each well and incubated for 30 min at room temperature. The reaction was stopped, and absorbance was read in an ELISA plate reader at 450 nm. The concentration of TGF-β1 was calculated according to a standard curve.

### Statistical analysis

One- and two-way analyses of variance (ANOVA) with post hoc analysis using Tukey's test using GraphPad Prism program ver. 5 (GraphPad Software, Inc., LA Jolla, CA, USA), and Student *t*-tests were used to evaluate the significance of the results. *P* values < 0.05 were considered statistically significant. Data were expressed as mean** ± **standard deviation (SD).

## Results

### *Expression of E-cadherin in *ex vivo* thyroid tissues*

**Fig. 1:**
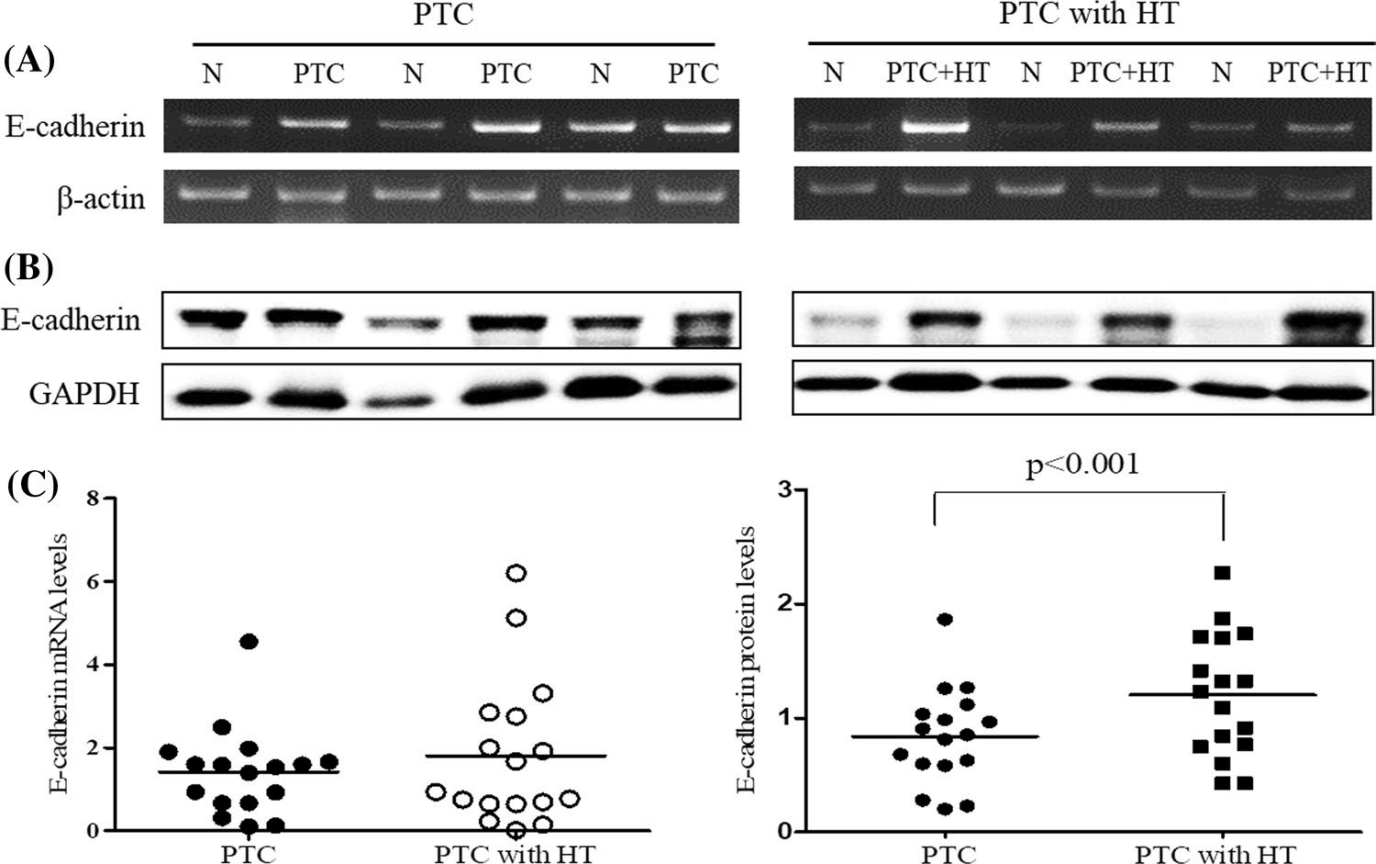
The expression of E-cadherin in thyroid tissues of patients with papillary thyroid cancer with and without Hashimoto’s thyroiditis. The expression of E-cadherin mRNA (**A**) and protein (**B**) was determined in normal and tumour tissues of the patients with PTC alone or PTC and HT by RT-PCR and Western blot, respectively. The images of RT-PCR (**A**) and Western blot (**B**) were presented representative bands of three patients with PTC or PTC with HT. The histogram of mRNA (**C**) and protein (**D**) levels was presented as mean (PTC and PTC with HT: *n* = 17 each) using densitometric analysis and comparing the results to levels in normal tissues. N, normal tissue; PTC, papillary thyroid cancer tissues; and PTC + HT, PTC tissues with Hashimoto’s thyroiditis

To investigate the expression of E-cadherin in the patients with PTC and HT or without HT, the expression of E-cadherin mRNA and protein was determined in the thyroid cancer tissues of each group by RT-PCR and Western blot, respectively. As a result, the expression levels of E-cadherin mRNA (Fig. 1A) and protein (Fig. 1B) were increased in the patients with PTC and PTC with HT compared with the normal group. In addition, the expression of E-cadherin protein in thyroid cancer tissues was significantly (*P* < 0.001) increased in the patients with PTC and HT compared with PTC alone (Fig. 1C).

### *Expression of N-cadherin in *ex vivo* thyroid tissues*

**Fig. 2:**
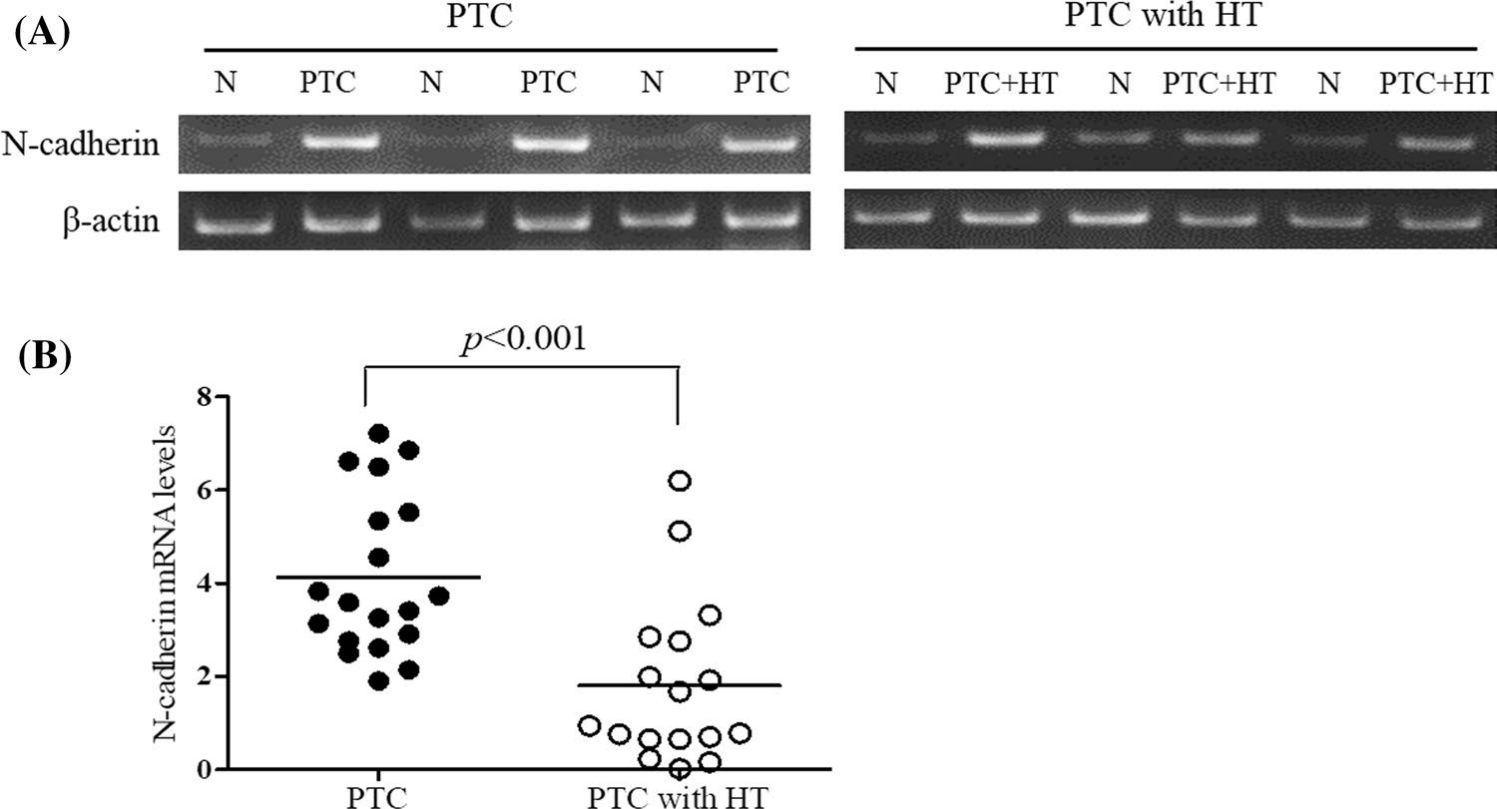
N-cadherin mRNA expression in thyroid tissues of the patients with papillary thyroid cancer with and without Hashimoto’s thyroiditis. **A** The expression of N-cadherin mRNA was determined in normal and tumour tissues of the patients with PTC alone or PTC and HT by RT-PCR. **B** The histogram was presented as mean expression levels (*n* = 17 each) using densitometric analysis and comparing the results to levels in normal tissues. N, normal tissue; PTC, papillary thyroid cancer tissues; and PTC + HT, PTC tissues with Hashimoto’s thyroiditis

To investigate the expression of N-cadherin, one of cadherin family, in the patients with PTC and HT or without HT, the expression of N-cadherin mRNA was determined in the thyroid cancer tissues by RT-PCR. As a result, the expression of N-cadherin was increased in the patients with PTC and HT compared with the normal group (Fig. 2A), and significantly (*P* < 0.001) decreased in the PTC with HT compared with PTC alone (Fig. 2B).

### Expression of E-cadherin and migration in thyroid cancer cells

**Fig. 3:**
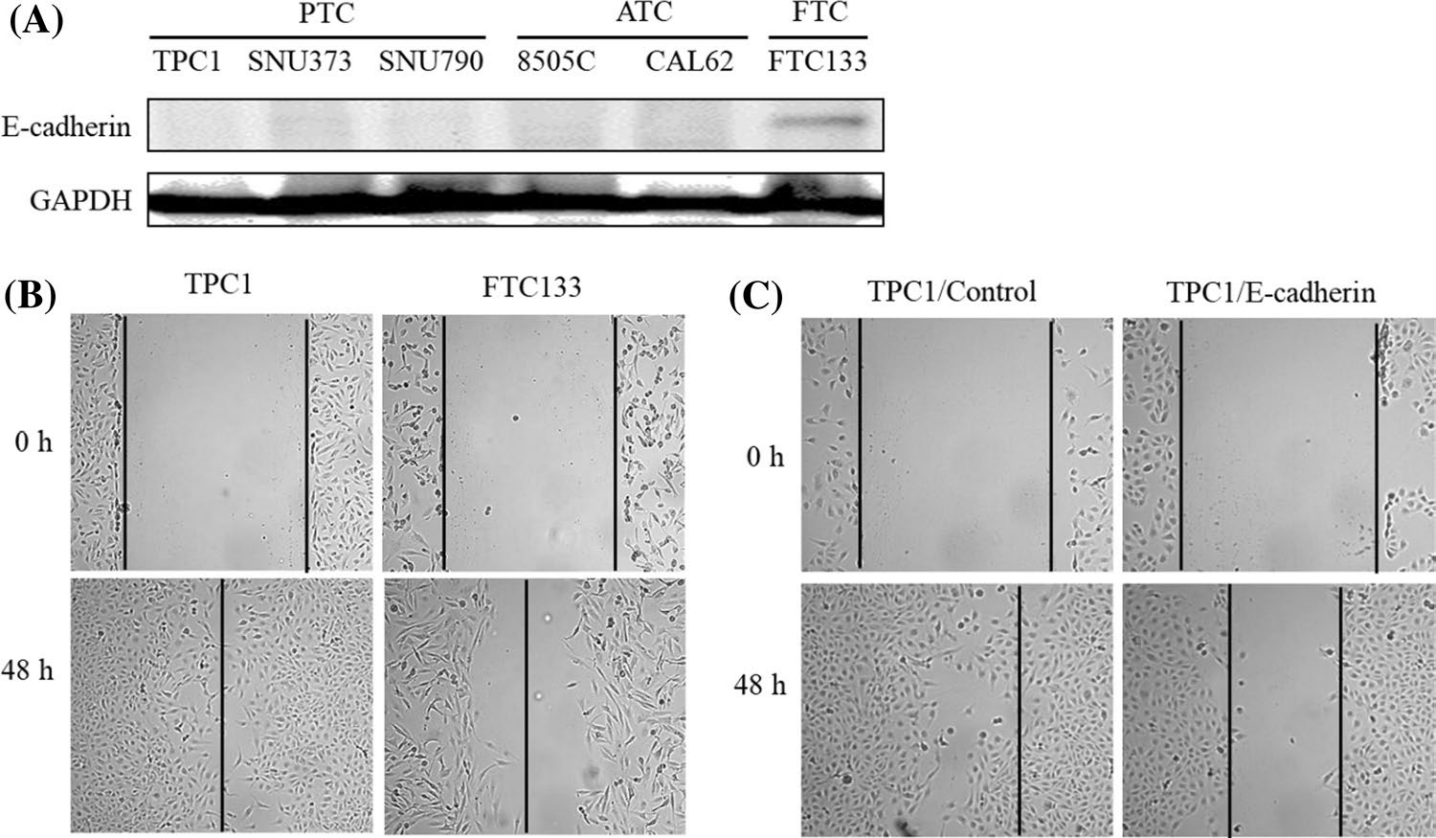
The expression of E-cadherin and cell migration in various cancer cell lines. **A** The expression of E-cadherin protein was determined in cells with papillary thyroid cancer (TPC), anaplastic thyroid cancer (ATC) and follicular thyroid cancer (FTC) by Western blot. **B** Cell migration was determined in TPC1 and FTC133 cells by wounded healing assay. **C** TPC1 cells were transfected with E-cadherin and then assayed cell migration. The morphology of cell growth was observed by microscope (original magnification ×100)

To investigate the expression of E-cadherin as a tumour suppressor in various thyroid cancer types, we measured the expression of E-cadherin protein in human papillary thyroid cancer cells (TPC: TPC1, SNU373 and SNU790), anaplastic thyroid cancer cells (ATC: 8505C and CAL62) and follicular thyroid cancer cells (FTC: FTC133) by Western blot. As a result, the expression of E-cadherin was only observed in FTC cells (Fig. 3A).

Next, to investigate the effect of the E-cadherin overexpression on migration in thyroid cancer cells, wound healing assays were conducted FTC133, TPC cells, and E-cadherin-overexpressing TPC1 cells. As a result, the cellular monolayer was re-established in the area of the initial “wound” after 48 h in normal TPC1 and FTC133 cells (Fig. 3B), but the overexpression of E-cadherin in TPC1 cells inhibited cell migration compared with control cells (Fig. 3C).

### *Expression of TGF-β1 in *ex vivo* thyroid tissues*

**Fig. 4:**
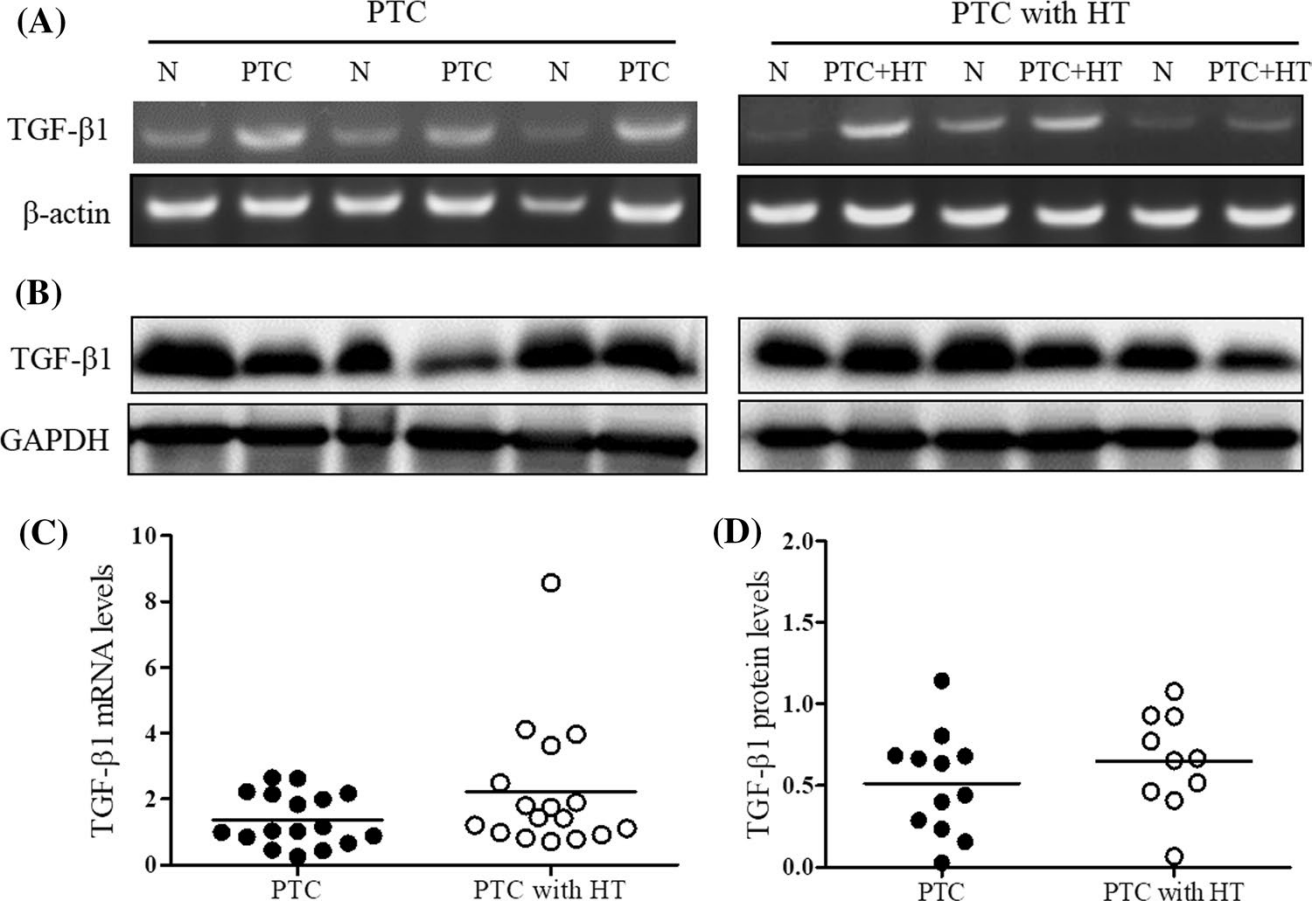
The expression of TGF-β1 1 in thyroid tissues of patients with papillary thyroid cancer with and without Hashimoto’s thyroiditis. The expression of TGF-β1 mRNA (**A**) and protein (**B**) was determined in normal and cancer tissues of patients with PTC alone or PTC and HT by RT-PCR and Western blot, respectively. The histogram of mRNA (**C**) and protein (**D**) levels was presented as mean expression levels (N; *n* = 17, PTC; *n* = 12, PTC + HT: *n* = 10) using densitometric analysis and comparing to levels in normal tissues. N, normal tissue; PTC, papillary thyroid cancer tissues; and PTC + HT, PTC tissues with Hashimoto’s thyroiditis

To investigate the expression of TGF-β1 as an activator of E-cadherin in the patients with PTC and those with HT, the expression of TGF-β1 mRNA and protein was detected in the thyroid cancer tissues of each group by RT-PCR and Western blot, respectively. As a result, the expression of TGF-β1 mRNA (Fig. 4A) and protein (Fig. 4B) was increased in the patients with PTC and in those with PTC and HT compared with the normal group. Also, the expression levels of TGF-β1 mRNA (Fig. 4C) and protein (Fig. 4D) were increased in the patients with PTC and HT compared with those with PTC alone.

**Fig. 5:**
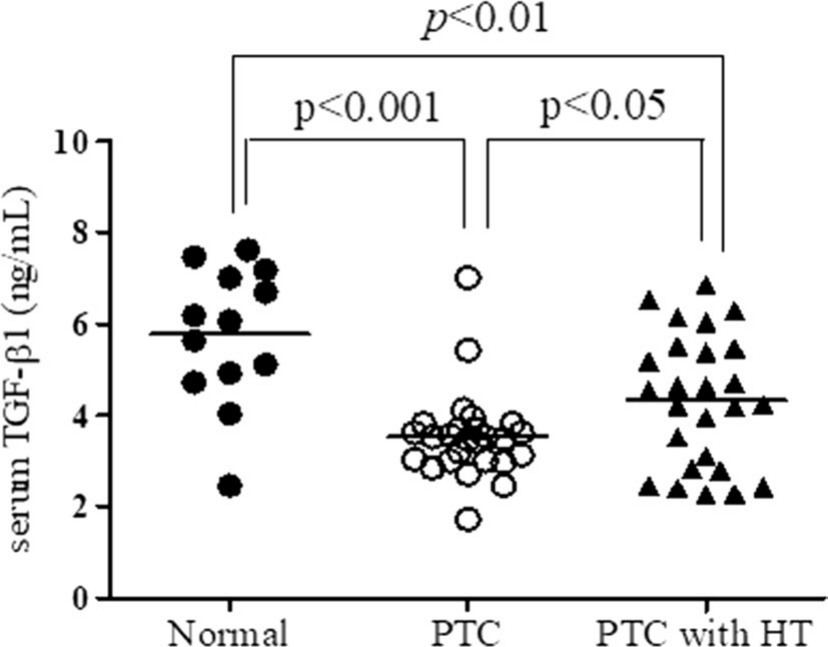
The levels of TGF-β1 in sera of patients with papillary thyroid cancer with and without Hashimoto’s thyroiditis. The concentration of TGF-β1 was measured in the serum of healthy controls (*n* = 13) and the patients with papillary thyroid cancer (PTC) alone (*n* = 26) or PTC and Hashimoto’s thyroiditis (HT) (*n* = 26) by ELISA

Next, we measured the plasma levels of TGF-β1 in the patients of each group by ELISA. As shown in Fig. 5, the plasma levels of TGF-β1 were significantly lower in the patients with PTC (*P* < 0.001) and PTC with HT (*P* < 0.01) compared with the normal group (Fig. 5). Meanwhile, the plasma levels of TGF-β1 were significantly (*P* < 0.05) increased in the patients with PTC and HT compared with those with PTC alone.

### Expression of ICAM-1 in ex vivo thyroid tissues

**Fig. 6:**
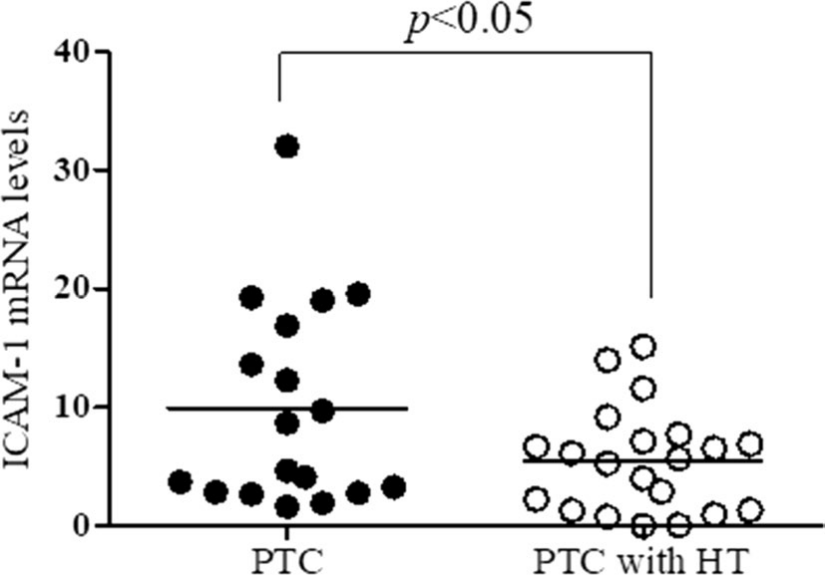
The expression of ICAM1 mRNA in thyroid tissues of patients with papillary thyroid cancer with and without Hashimoto’s thyroiditis. The expression of ICAM1 mRNA was determined in normal and cancer tissues of patients with papillary thyroid cancer (PTC) alone or PTC and Hashimoto’s thyroiditis (HT) by real time-PCR. The histogram was presented as mean expression levels (PTC, *n* = 18; and PTC with HT, *n* = 21) relative to levels in normal tissues

To investigate the expression of ICAM-1 as a carcinogenesis mediator in the patients with PTC and those with HT, the expression of ICAM-1 mRNA was detected in the thyroid cancer tissues of each group by RT-PCR. As a result, the expression of ICAM-1 mRNA was significantly decreased (*P* < 0.05) in the patients with PTC with HT compared with PTC alone (Fig. 6).

## Discussion

The relationship between PTC and HT remains unclear because it is controversial whether HT is induced secondarily to the neoplasm or whether HT predisposes the patient to develop thyroid cancer. In previous studies, the coexistence of PTC with HT has been associated with smaller tumour size, younger age, female predominance, reduced risk of recurrence, lower incidence of extra-nodal extension, lymph node metastasis ratio, early stage disease, and better prognosis compared with PTC alone [14–18]. Although autoimmune mechanisms have been suggested to explain the improved prognosis of patients with PTC and HT compared to PTC alone, the underlying mechanism remains unclear.

In this study, we investigated the correlation between the expression of E-cadherin and the aggressiveness of PTC in patients with concomitant PTC and HT using the tissues samples. In our study, the expression of E-cadherin was higher in samples from patients with PTC and HT than in normal volunteers or patients with PTC alone. This result suggests that the presence of concomitant HT decreases the aggressiveness of PTC by positively regulating E-cadherin expression. On the other hand**,** it was reported that E-cadherin expression is more associated with histopathological type of thyroid cancer than with the metastatic potential of tumours, and is not a predictor of extra-thyroidal growth of thyroid cancers [13]. In histological observation, the loss of E-cadherin expression may be involved in regional lymph node metastasis and in malignant potential of thyroid neoplasm [19,20]. Several studies showed that N-cadherin can inhibit cell growth [21, 22], and its overexpression was correlated with poor histological differentiation in tumours [23]. In our study, the expression of N-cadherin was significantly decreased in the samples from patients with PTC and HT compared with those with PTC alone. This may suggest that the lower expression of N-cadherin in the samples from patients with PTC and HT than in those from patients with PTC alone may relate to suppression of N-cadherin in poor histological differentiation. However, further studies such as microscopic observation with immunohistochemistry are required to better understand E-cadherin roles in development and tumorigenesis of PTC with HT. As far as we know, the difference of N-cadherin expression between patients with PTC and HT and those with PTC alone has not been reported.

TGF-β has three different isoforms, including activins, bone morphologic proteins, and growth differentiation proteins [24], and achieves intracellular signalling via phosphorylation of the Smad2 and Smad3 proteins and translocate to the nucleus to regulate gene expression. Recent evidence suggests that the thyroid gland expresses TGF-β under physiologic conditions to regulate thyroid growth and function, and TGF-β1 cell signalling is important in the development of PTC [25]. In this study, we demonstrated that TGF-β was expressed more in the thyroid tissues of patients with PTC and HT than in those with PTC alone. Also, the plasma level of TGF-β was increased in patients with PTC and HT compared with those with PTC alone. In contrast to the thyroid tissues, interestingly, the plasma level of TGF-β was lower in patients with PTC or PTC and HT compared with the normal group. Recently, TGF-β has been shown to play a role in PTC; however, to date few studies examining the potential diagnostic marker of TGF-β for PTC have been completed [25]. Evidence suggests that TGF-β may act as an autocrine growth inhibitor on thyroid follicular cells, and decreased release of and responsiveness to TGF-β may be cofactors in with the pathogenesis of iodine-deficient non-toxic goitre [26].

ICAM-1 is transmembrane glycoprotein receptor belonging to the immunoglobulin superfamily of adhesion molecules and normally expressed on the surface of various types of cells [27]. ICAM-1 has been found to be upregulated in many cancers and has been implicated in carcinogenesis [28]. ICAM-1 expression is upregulated in patients with PTC, and correlated with aggressiveness tumour features such as BRAF^V600E^ mutation, extra-thyroidal extension, and lymph node metastasis in thyroid cancer progression [29]. Similar to the previous studies, we confirmed that the expression of ICAM-1 gene was lower in the patients with PTC and HT than in those with PTC alone. This result suggests that HT may have a role in the downregulation of the ICAM gene in PTC and HT, but further studies evaluating the presence of ICAM-1 in PTC and PTC with HT may be warranted.

## Conclusions

In conclusion, we confirmed that the expression of E-cadherin and the production of TGF-β1 are higher in patients with PTC and HT compared with those with PTC alone, but the expression levels of N-cadherin and ICAM-1 are lower in patients with PTC and HT. These results indicate that HT may have an unaggressive role in regional lymph node metastasis and malignancy potential of thyroid neoplasm through upregulation of E-cadherin and TGF-β1 expression and downregulation of N-cadherin and ICAM-1 expression. However, further investigation is necessary to elucidate the mechanisms underlying the relationship between HT and the expression of these cytokines.

## Data Availability

All data used to support the findings of this study are available from the corresponding authors.
